# Reperfusion of Pulmonary Arteriovenous Malformations Treated by Catheter Embolization †

**DOI:** 10.3390/jcm13247812

**Published:** 2024-12-20

**Authors:** Bianca Gulich, Arno Buecker, Guenther Schneider

**Affiliations:** Clinic of Diagnostic and Interventional Radiology, Saarland University Medical Center, 66421 Homburg, Germany; arno.buecker@uks.eu (A.B.); dr.guenther.schneider@uks.eu (G.S.)

**Keywords:** hereditary hemorrhagic telangiectasia (HHT), arteriovenous malformations, embolization

## Abstract

**Objective:** The aim of this study was to evaluate patients with hereditary hemorrhagic telangiectasia (HHT) for the potential reperfusion of pulmonary arteriovenous malformations (PAVM) treated by catheter embolization using coils or embolization plugs and to analyze causes of possible reperfusion in order to further improve treatment. **Methods:** This retrospective study analyzed the data of 345 patients who underwent screening for pulmonary arteriovenous malformations in cases of suspected or confirmed HHT (Osler’s disease). Of these, 118 patients with PAVM that underwent catheter embolization and had at least one follow-up study were included in our study and evaluated for potential reperfusion. Screening and follow-up for the detection of PAVM was performed by dynamic and high-resolution contrast-enhanced magnetic resonance angiography (MRA). The average follow-up time was 6.2 years. **Results:** Reperfusion was detected in 43 of 118 patients at follow-up. Thirty-five of these patients showed a recanalization of the treated vessel and in eleven patients the formation of collateral vessels resupplying the PAVM were identified as the cause of reperfusion. The average time between embolization and detected reperfusion was 5.6 years. The recanalization of both coils and plugs was observed. The recanalization of coils could be attributed in most cases to an insufficient packing density of the implanted coils. In addition, an enlarged diameter of the feeding artery was confirmed as a risk factor for reperfusion. **Conclusions:** As the reperfusion of embolized pulmonary arteriovenous malformations can occur after a long time interval post-treatment, regular lifelong follow-up studies after embolization are essential to detect reperfusion at an early stage and avoid serious complications like a brain abscess or stroke through prompt re-embolization. After coil embolization, attention should be paid to sufficiently dense packing to achieve adequate and permanent occlusion.

## 1. Introduction

Osler’s disease is an autosomal dominantly inherited disease with an estimated prevalence of between 1:5000 and 1:21,000 [1,2,3]. Symptoms of the disease are mainly nosebleeds and telangiectasias, with the telangiectasias typically appearing after the first episode of epistaxis [4,5,6,7]. However, as it is a systemic disease, AV fistulas can occur anywhere in the body, with manifestations in lung, liver and brain being the most common and clinically important [8]. If the case of arteriovenous malformations in the lung (PAVM), this can lead to serious complications including stroke and intracerebral abscesses, with the latter often being the first manifestation of the disease [9,10]. Catheter-based embolization is therefore recommended for the treatment of PAVM in order to prevent complications and improve possible hypoxemia [11,12]. Reperfusion is a known problem after embolization which again poses the risk of right-to-left shunting and the above mentioned complications [13,14]. Various causes of reperfusion such as recanalization and collateral formation have been described in the literature, but only a small amount of further information is available. The aim of this study is to systematically evaluate these and other causes of reperfusion of patients with HHT in order to optimize treatment strategies for pulmonary arteriovenous malformations [13,15].

## 2. Materials and Methods

### 2.1. Patient Selection

**Figure 1:**
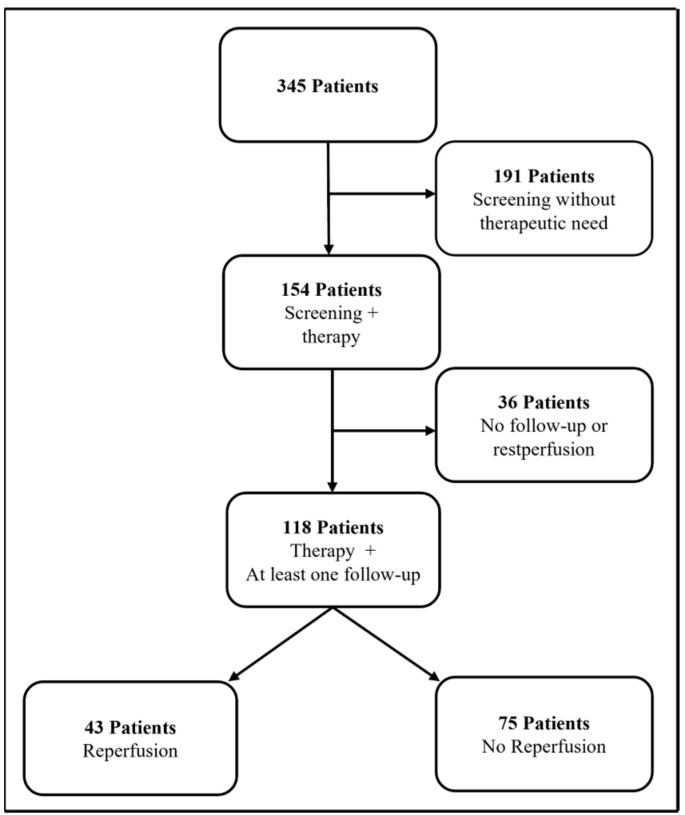
Distribution of patients with screening for newly developed or reperfused PAVM at the Medical Center for Diagnostic and Interventional Radiology of Saarland University.

In this retrospective study, 345 patients were screened for visceral manifestations of the disease, focusing on the brain, lung and liver. Patients with suspected and proven Osler’s disease were included in the study. PAVMs were detected in 154 patients, which required catheter-based embolization using coils and/or plugs due to a supplying artery size diameter of ≥2 mm [9]. In total, 118 of these patients had at least one follow-up; this patient cohort formed the basis of our study. The majority of patients (98) were embolized in our department. A total of 20 patients were transferred to our clinic for further treatment after external embolization, of which reports of the treatment performed elsewhere were only available for 4 patients. In 43 patients, the reperfusion of treated malformations was detected at follow-up. The remaining 75 patients had no reperfusion at follow-up which formed our comparison group (Figure 1 shows a flow chart of patient allocation). The images from the screening and follow-up were analyzed by a single experienced senior physician (G.S.) and assessed with regard to their relevance for therapy. If (re-) embolization was necessary, it was performed shortly after the screening.

### 2.2. Imaging

Magnetic resonance angiography (MRA) and Digital subtraction angiography (DSA) images of the patients were used to diagnose the status of PAVM. In the patients with a detected reperfusion in the follow-up examinations, the images of the re-embolization were also evaluated with regard to possible causes of reperfusion. Both the screening and the follow-up checks were carried out using contrast-enhanced magnetic resonance imaging (1.5 Tesla, Magnetom Aera, Siemens Healthcare, Erlangen, Germany). Image sequence parameters are given in Table 1. A contrast agent bolus of 0.025 mmol/kg bw (MultiHance^®^, Bracco Imaging Deutschland GmbH, Constance, Germany) was first injected to create a dynamic MR angiography and then a bolus of 0.075 mmol/kg bw (MultiHance^®^, Bracco Imaging Deutschland GmbH, Constance, Germany) was administered to perform a high-resolution MR angiography. The original images of externally treated patients were available in one case.

### 2.3. Catheter Embolization Procedure and Follow-Up

For the catheter embolization of PAVM, the right common femoral vein was first punctured using the Seldinger technique and then a sheath with a size of 8 F was inserted. Afterwards, the common pulmonary artery was accessed using a 5 F pigtail catheter (SUPERTORQUE™ MB, Cordis, Norderstedt, Germany), and for the selective catheterization of the (sub-) segmental pulmonary arteries, either a Cook White Lumax catheter (Cook Medical LLC, Bloomington, IN, USA) or a coaxial system consisting of a Neuron MAX 6 F Long Sheath (outer diameter 8 F) and Neuron 6 F Select Catheter (H1, 6F, Tip Diameter 5F) (Penumbra Europe GmbH, Berlin, Germany) were exchanged over a Rosen guidewire (Cook Medical LLC, Bloomington, IN, USA). After contrast medium injection, the number and diameter of feeding arteries were evaluated for further treatment planning, whereby the embolization material was placed as close as possible to the nidus without being placed inside. At our department, Nester^®^ embolization coils (Cook Medical LLC, Bloomington, IN, USA) and Amplatzer™ Vascular Plug II and 4 (Abbott Medical, Plymouth, MN, USA) were predominantly used for this purpose. The diameter of the initially inserted coils was chosen to be around 50% and that of plugs around 25% larger than the diameter of the feeding artery in order to prevent the migration of the embolization material into the systemic circulation. After embolization angiography was performed to ensure the total occlusion of the PAVM and proper positioning of the embolization material. Further, a cranial magnetic resonance imaging was performed 4 hours after embolization in order to rule out peri-interventional ischemia. Subsequently, a follow-up visit was carried out three months after embolization and again one and five years later to detect reperfusion or newly developed PAVM. Due to the lack of reports from most externally treated patients, no statement can be made about all external embolization procedures or materials.

### 2.4. Statistical Analysis

Statistical analysis was performed using IBM SPSS Statistics 28.0 (IBM Deutschland GmbH, Böblingen, Germany) program for the following parameters: in order to check whether the age at embolization was related to a later reperfusion, the Kolmogorov–Smirnov test was first used to check whether a normal distribution was present. Once this was confirmed, a t-test was performed. A chi-square test was used to check the gender distribution of patients with and without recanalization and for the distribution of the therapy-relevant PAVM within the lung. In addition, a Mann–Whitney U test with independent samples was performed to statistically test the diameter of the feeding artery between the two patient groups (a *t*-test could not be performed due to non-normally distributed values). For testing the difference between coils with the thickness of 0.035 inch and coils with a lower thickness, a two-sided chi-square test was performed using the NCSS 2019, v19.0.1 statistical software (NCSS, LLC, East Kaysville, UT, USA). The significance level was set at a *p*-value of *p* ≤ 0.05.

## 3. Results

### 3.1. Reperfusion Rates

A total of 394 PAVMs were embolized in 118 patients. Of these, 43 patients showed reperfusion at follow-up, with a total of 72 PAVMs being reperfused. In 35 patients, the recanalization of the treated PAVM was identified as the cause of reperfusion and in eleven patients the formation of collateral circulation was identified, which continued to perfuse the PAVM. The 62 recanalized PAVMs correspond to 15.7% and the 12 PAVMs reperfused by collateral circulation correspond to 3% of the total number of embolized PAVMs. In two malformations, reperfusion could be identified as a result of both recanalization and collateral formation.

**Figure 2:**
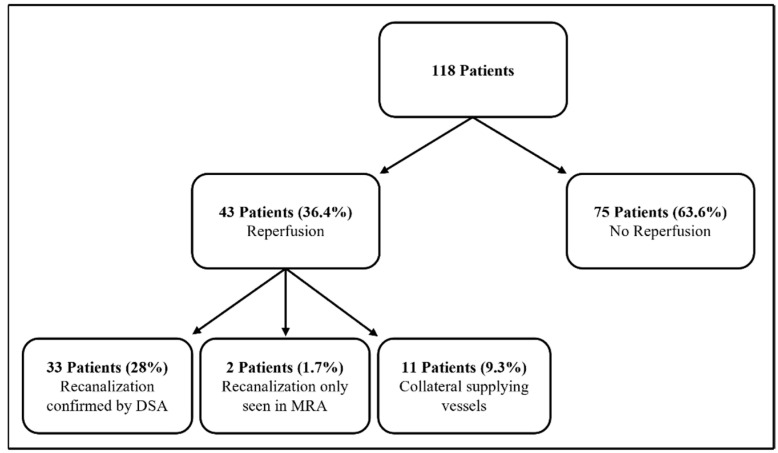
Flow chart showing a detailed distribution of the patients with reperfusion (with share of the total collective).

In 33 of the 35 patients with detected recanalization in MRA, reperfusion was confirmed in a subsequent DSA. In two patients, the diameter of the feeding artery was ≤2 mm. Therefore, no indication for treatment was seen and no catheter angiography was performed [9]. Figure 2 shows a flow chart of the patients with respect to reperfusion.

### 3.2. Follow-Up Period

The average follow-up period of our patient population was 6.2 years ranging from 3 to 276 months (23 years). However, the follow-up period after therapy could only be determined for 102 of the 118 patients, as only 98 of the 118 patients were embolized at our center. A total of 20 patients were transferred to our clinic for further treatment after external embolization. The reports on the performed examinations were only available in four external embolized cases.

### 3.3. Time Between Embolization and Detected Reperfusion/Re-Embolization

**Figure 3:**
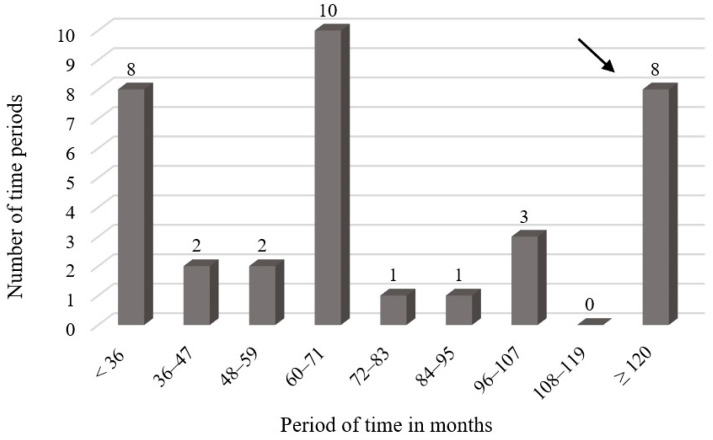
Distribution of time periods between initial treatment and re-embolization of the patients with known embolization date.

In addition, the period between embolization and the first described reperfusion was determined using MRA. This includes patients with recanalization and collateral formation after embolization and was determined on the basis of the 22 patients treated at our clinic and the 4 treated externally with a known embolization date. A period of 5.6 years was determined with a range from 3 to 168 months. In this context, we further determined the time between embolization and the new treatment of the recanalized malformations in patients with DSA. This could be evaluated for 20 of the 33 patients, with a total of 33 recanalized malformations. In one patient, the recanalization of the same malformation occurred a total of three times, resulting in a total of 35 time spans. The arithmetic mean recanalization time was 6.25 years, with values ranging from 3 to 168 months. Figure 3 shows a detailed breakdown of recanalization times.

### 3.4. Age Distribution of Patients That Underwent Embolization

With regard to patients` age at embolization, the following findings were obtained: in the patients with recanalization and subsequent re-embolization, the age at the embolization of malformations that were found to be recanalized could be determined for 20 of 33 patients. One patient underwent embolization at two different times, resulting in a total of 21 values. The arithmetic mean age at embolization of the patients with recanalization was 42.2 years, with a standard deviation of 14.6 years and a range of 4 to 67 years. In patients without reperfusion, the arithmetic mean was 43.2 years with a standard deviation of 16.8 years and a range of 8 to 77 years. In order to evaluate whether the age at embolization has an influence on subsequent recanalization, the age distribution in both groups was first checked for a normal distribution using the Kolmogorov–Smirnov test. After the confirmation of the normal distribution, we performed a t-test afterwards. With a *p*-value of *p* = 0.814, this was above the defined significance level of *p* ≤ 0.05, meaning that there was no statistically significant difference between the age of patients with embolization of later recanalized malformations and the age of patients without later reperfusion. This suggests that the growth of embolized PAVMs in childhood does not lead to an increased reperfusion rate.

### 3.5. Gender Distribution of Patients That Underwent Embolization

In total, 22 patients with recanalization and subsequent angiography were female and 11 patients were male. This resulted in a female-to-male ratio of 2:1. In the comparison group, 53 patients were female and 22 were male, which corresponds to a female-to-male ratio of 2.4:1. To investigate whether reperfusion occurs more frequently in one gender, the gender distribution between the two groups was analyzed using the chi-square test. However, with a *p*-value of *p* = 0.678, no statistically significant difference was found in the gender distribution of the two groups, which indicates that reperfusion does not occur more frequently in a particular gender. The female-to-male ratio in both groups also shows that therapy-relevant PAVM in adults occurs more frequently in women.

### 3.6. Recanalized Feeding Arteries Depending on the Embolization Material

**Figure 4:**
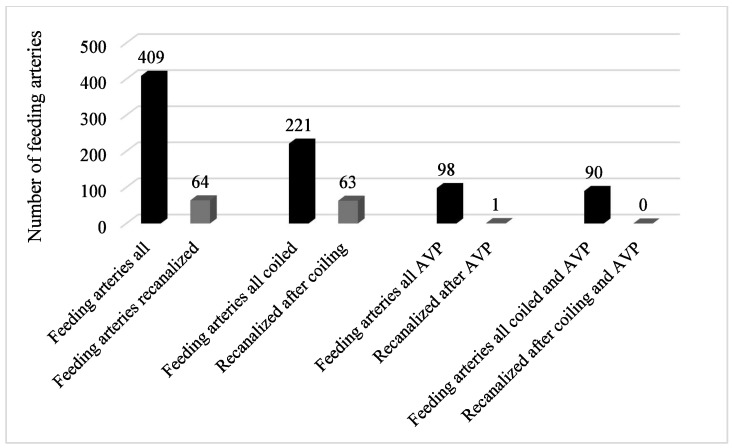
Distribution of recanalized feeding arteries depending on embolization material.

In the patients with and without recanalization and subsequent DSA, a total of 409 feeding arteries were embolized in 359 malformations. Of these 409 feeding arteries, 64 were identified as recanalized during the follow-up period. Of these 64 feeding arteries, 63 were initially embolized by coils and the recanalization of one Amplatzer vascular plug 4 (AVP) was observed. Figure 4 shows the distribution of embolization materials used to occlude the feeding arteries.

### 3.7. Size of the Feeding Artery

**Figure 5:**
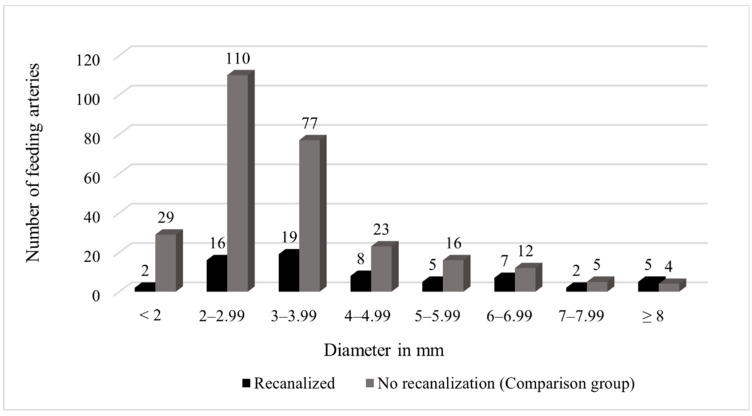
Distribution of the diameter of the feeding arteries in both groups.

The diameter of the feeding artery was determined for further evaluation. In the 33 patients with recanalization and DSA, an arithmetic mean of 4.4 mm with a standard deviation of 2.2 mm and a range of 1.6 mm to 11.8 mm was determined. Within the comparison group, the diameter of 276 of the 282 occluded feeding arteries could be evaluated. The arithmetic mean was 3.4 mm with a standard deviation of 1.5 mm whereby the range extended from 1.1 mm to 10.5 mm. Figure 5 shows the distribution of the measured values. The dependence of recanalization on vessel diameter was tested using the Mann–Whitney U test with independent samples yielding a *p*-value of *p* < 0.001, which indicates that an increased diameter of the feeding artery is a risk factor for reperfusion.

### 3.8. Distribution of Therapy-Relevant PAVM Within the Lung

**Figure 6:**
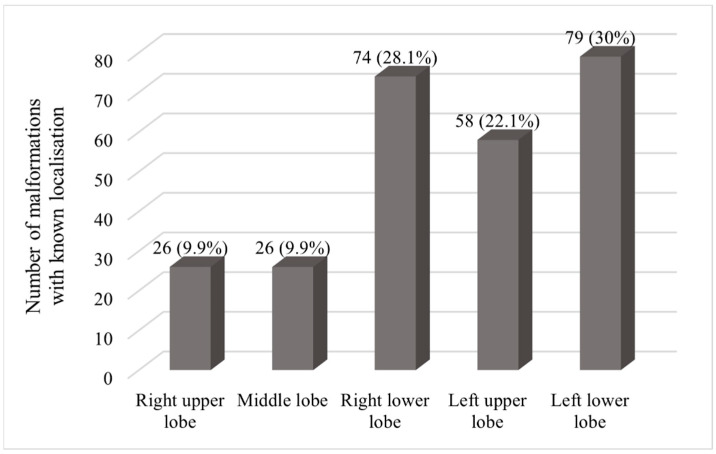
Distribution of PAVM with known localization within the lung.

Within the patients with recanalization and DSA as well as the comparison group, the distribution of treatment-relevant PAVMs across the different lung lobes was examined. Depending on the supply area of the feeding artery, these were allocated to the right upper, middle or lower lobe or to the left upper or lower lobe. In the patients with recanalization and DSA, the localization could only be determined for 64 of the 105 treated PAVMs. In patients without reperfusion, the location of only 199 of the embolized PAVMs could be evaluated. Figure 6 shows the distribution of the 263 identifiable PAVMs. In this context, a one-dimensional chi-square test was also performed to statistically test the distribution of treatment-relevant PAVM. This resulted in a *p*-value of *p* < 0.001, which shows a statistically significant difference in the size of the different groups and thus an increased incidence of therapy-relevant PAVM in the basal lobes of the lungs.

### 3.9. Evaluation of Recanalized Feeding Arteries with Regard to the Cause of Reperfusion

**Figure 7:**
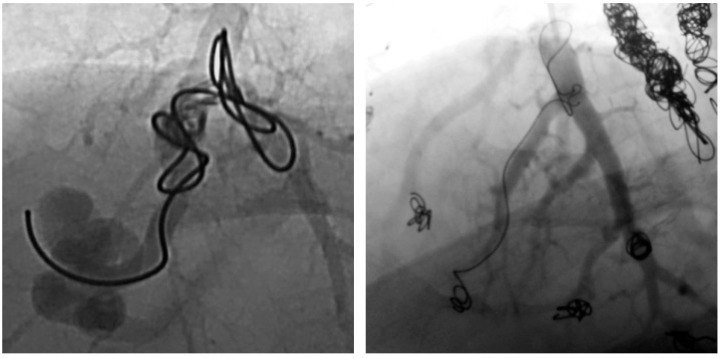
Presentation of two PAVM with insufficient packing density of implanted coils.

**Figure 8:**
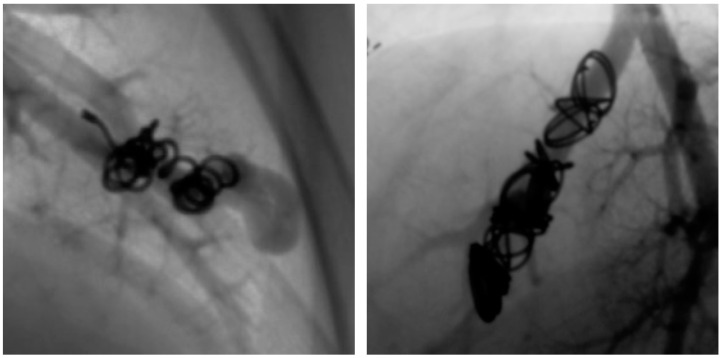
Presentation of two PAVM with spiral creation of implanted coils at the arterial wall.

Furthermore, angiography images were examined for possible causes of reperfusion during re-embolization. In a total of 64 recanalized AV shunts, recanalization could be attributed to inadequate packing density of the implanted coils in 52 feeding arteries. Two examples are shown in Figure 7. In 15 of the 52 recanalized feeding arteries, we can further see a positioning of the embolization material on the wall of the vessel but not in the center (Figure 8).

**Figure 9:**
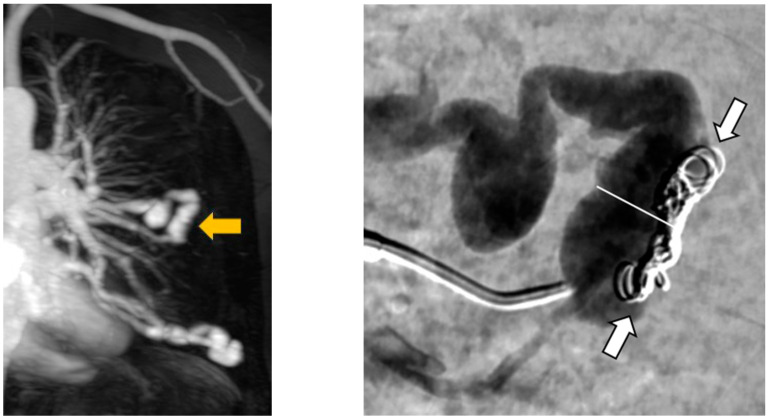
MRA shows a dilatation of the feeding artery at the implantation site (**left**) and confirmation by an implemented DSA (**right**).

The perfusion of PAVM was observed in two externally embolized malformations, which were initially embolized by detachable coils in the aneurysm sac. In another patient, reperfusion could be attributed to an increase in the size of the feeding artery. During the initial embolization at the age of four years, the diameter was 1.5 mm and seven years later a diameter of 2.1 mm was determined during re-embolization. In another patient, the dilatation of the feeding artery in the area of the implanted embolization material was observed. This was already seen in the MRA performed and confirmed in a subsequent angiography. Furthermore, the fixation of the implanted coils in the arterial wall was evident in the DSA series. Figure 9 shows both the screening and the angiography images, with the open arrow in the MRA image showing the dilation of the feeding artery and the white arrows in the DSA image indicating the fixation of the implanted coils. The white line also emphasizes the dilatation of the feeding artery. It should also be emphasized that the embolization material completely filled the feeding artery during initial therapy and that the complete occlusion of the vessel was achieved.

**Figure 10:**
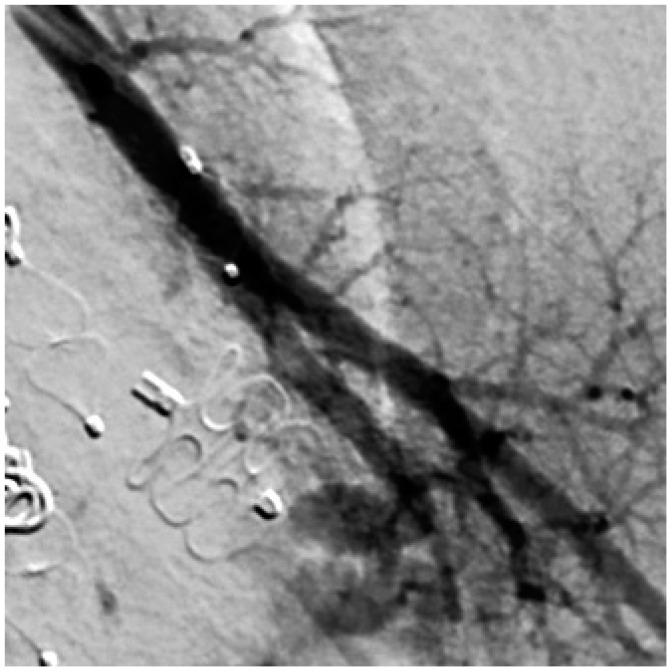
DSA of a recanalized AVP 4.

In addition, the recanalization of an Amplatzer™ vascular plug 4 was observed in one malformation. At follow-up visits after three months, one year and four years, this AV shunt was still occluded and recanalization was detected by MRA at a follow-up visit five years and ten months after initial embolization (Figure 10).

In another patient, the recanalization of a malformation occluded by tungsten coils was detected, with the recanalization being identified in 2008. It was not possible to determine the exact date of embolization, as the embolization took place before the start of digital recording of examination reports in 2002, but a minimum period of six years between embolization and recanalization can be assumed. The patient could not recall the year of embolization. It is already known for tungsten coils that corrosion can occur, so this is a possible recanalization mechanism in this patient. In four patients with a total of five recanalized malformations, no obvious cause of recanalization could be determined. In one of these four patients, however, it is striking that the same malformation had to be re-embolized a total of three times.

**Figure 11:**
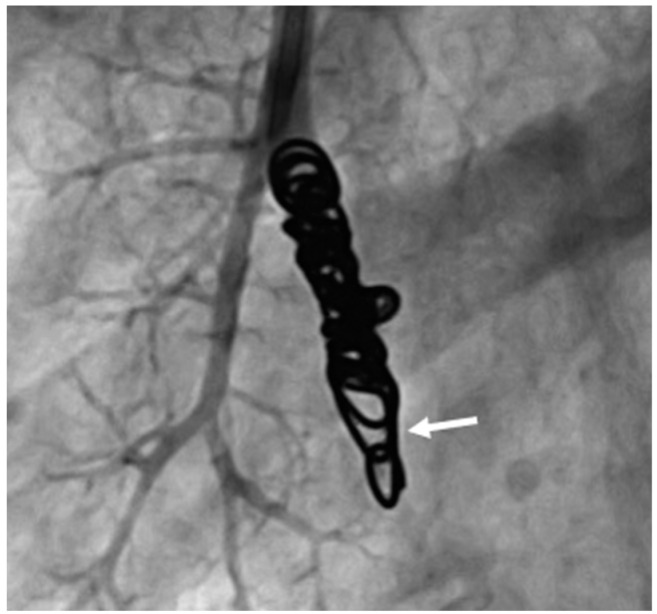
Nester^®^ Embolization Coils with a thickness of 0.035 inch.

**Figure 12:**
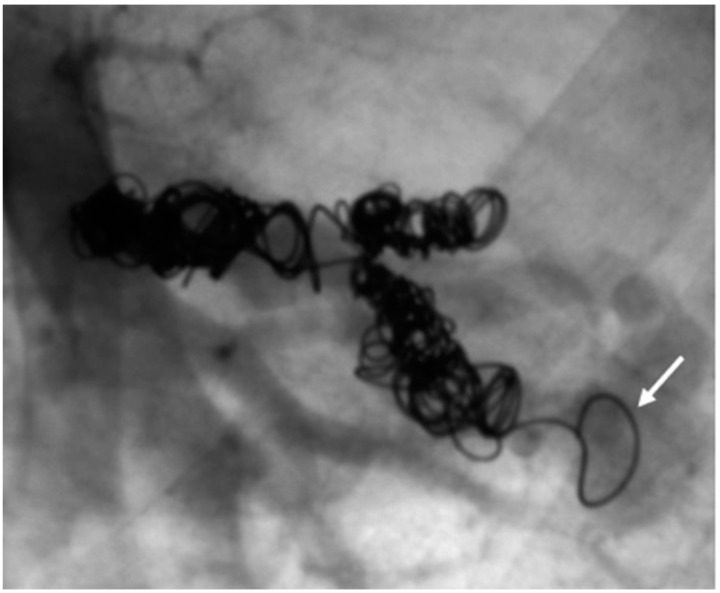
Coils of a patient with external treatment and which appear thinner than the Nester^®^ Embolization Coil.

When assessing the implanted embolization material, it was also noticed that some of the coils in the DSA appeared thinner than others. Figure 11 shows a Nester^®^ Embolization Coil from Cook Medical, which is available in a thickness of 0.035 inch and is regularly used in our department. In ten patients with a total of 29 recanalized AV shunts, the implanted coils appeared thinner than those used at our department, with these accounting for 45.3% of the total number of recanalized AV shunts. An example is therefore shown in Figure 12. In this context, a possible correlation between the use of coils with a lower thickness and a possible increased recanalization rate after embolization was discussed.

Therefore, we further evaluated the embolization materials in PAVMs with and without recanalization. For the patients without recanalization, 79 AV shunts have been embolized only by coils with a thickness of 0.035 inch and four other feeding arteries with a thickness lower than 0.035 inch. For the patients with recanalization, evaluation was more difficult due to the fact that only 16 of them have been treated at our department. In those cases that were embolized elsewhere, it was only possible to visually distinguish between a coil with the known thickness of 0.035 inch and embolization material with a lower diameter. To evaluate a possible correlation between the thickness of coils used for embolization and later reperfusion, we performed a two-sided chi-square test showing a *p*-value of *p* < 0.001, indicating that the coils with lower thickness seem to reperfuse more often than coils with a thickness of 0.035 inch.

The reperfusion of malformations by collaterals was identified in eleven of the embolized patients. For the patients treated in domo, the period between embolization and reperfusion could be determined: in two patients, this was identified at the first follow-up appointment after three months. In three further patients, this was identified at the second follow-up appointments 15 and 16 months after embolization. Collateralization between two pulmonary arteries occurred in ten of the eleven patients, whereby one patient showed collateralization of two different malformations.

**Figure 13:**
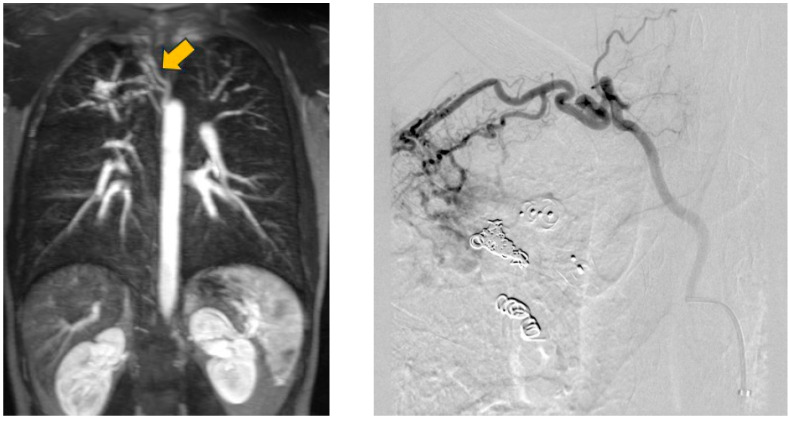
MRA (**left**) with presentation of collateral vessels by the systemic circulation supplying the PAVM (open arrow) and confirmation by DSA (**right**).

**Figure 14:**
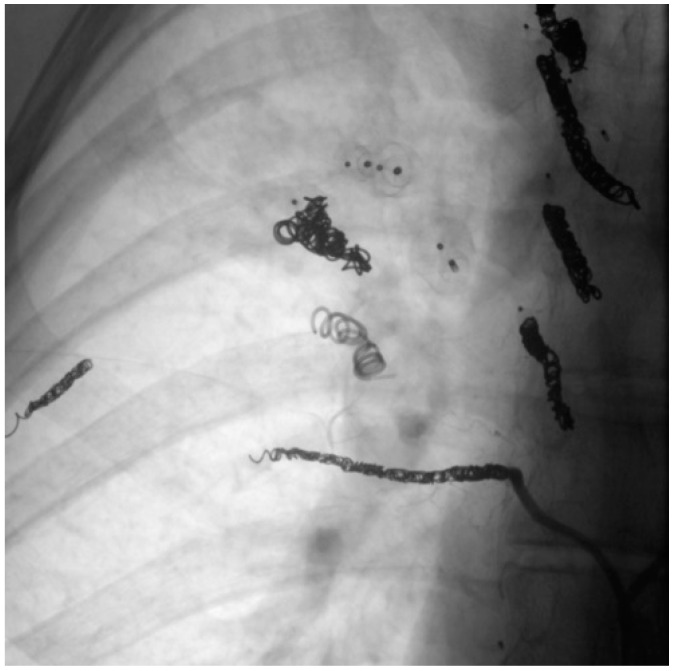
DSA with presentation of embolized collaterals between intercostal and pulmonary arteries.

**Figure 15:**
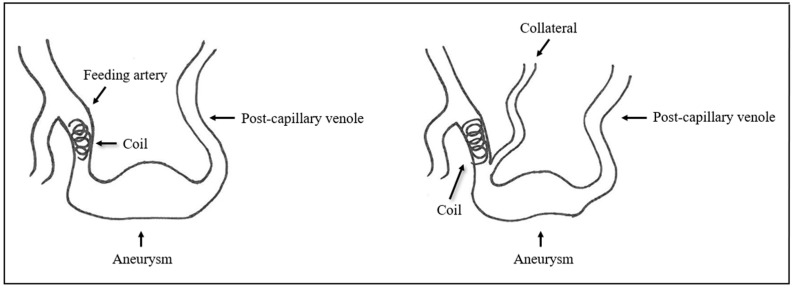
Schematic representation of collateral formation after embolization.

In another patient, however, the formation of collaterals between the intercostal and pulmonary arteries in the sense of aortopulmonary collaterals was detected (Figure 13 and Figure 14). Figure 15 also schematically shows the reperfusion mechanism of embolized PAVM with collateral formation.

## 4. Discussion

Reperfusion after embolization can lead to serious complications for patients, making follow-up and early diagnosis a mainstay of correct treatment. These complications include brain abscess and stroke formation due to paradoxical embolism, which can have far-reaching consequences for those affected, potentially causing visual disturbances and paresis which can affect them for the rest of their lives [9,16,17,18]. Due to the mechanism of origin, this also predominantly affects younger patients, so close follow-up monitoring after embolization is even more important [9,19]. Our study was performed to identify the causes of reperfusion in order to further improve the treatment of PAVM. With 43 patients out of 118 suffering from reperfusion, the percentage of reperfusion (36.4%) is higher than reported in other studies: In a study by Pollak et al., 7.6% of treated patients showed reperfusion or persistence after therapy [13]. In another study by Lee et al., recanalization after therapy was identified in 9% of treated patients [20]. This discrepancy between our and the other two studies is probably due to two reasons. On the one hand, the mean follow-up period of 6.2 years in this study is longer than 2.9 and 4.7 years of the other two studies. A study by Shimohira et al. already showed that the reperfusion rate increases with longer follow-up [21]. On the other hand, only 23 of the 43 reperfused patients were embolized at our clinic, and 20 patients with reperfusion were transferred to our clinic for further follow-up after external treatment, which is why this leads to a distortion of the reperfusion rate and the value for the patients treated internally is lower. The proportion of 15.7% of recanalized PAVMs is in the same order as described in the literature, where recanalization rates of between 5% and 19% are reported [14,22]. The present study also confirmed that recanalization occurs more frequently than collateral formation after embolization [14,23]. A recanalization occurs through a re-opening of the previously occluded part of the feeding artery so that there is again a blood flow through this artery and especially through the implanted embolization material. The reperfusion mechanism of collateral formation consists of bypassing the occluded part of the feeding artery by new vessels, with the reperfusion of the malformation distal of the embolization material (Figure 15).

At 5.6 years, the period between initial therapy and re-embolization was significantly longer than the periods described in other publications. Two studies described the recanalization of a total of three Amplatzer™ Vascular Plugs after 7 weeks and 36 months [24,25]. Another study reported the reperfusion of six malformations after more than two years, although these were still occluded at a follow-up one year after treatment [15]. However, the follow-up period of 74 months in our study was also longer than in the other studies (case report, 28 months and 41 months). Furthermore, only part of the patient cohort adhered to the recommended follow-up intervals, with many patients attending the screening significantly later than specified, which also contributes to a longer reperfusion period. Nevertheless, our data show that reperfusion can still occur long after the initial therapy, which is why the lifelong monitoring of patients is necessary for the early detection of reperfusion.

The age of the patients undergoing embolization was also determined. With a calculated arithmetic mean of 42.2 years for patients with recanalization and 43.2 years for patients without reperfusion, this information is consistent with the values in other publications. A study by Cottin et al. describes an average age of 42 years at the diagnosis of a treatment-relevant PAVM [4]. Furthermore, increasing numbers of diagnoses are reported in the fifth and sixth decade of life [26]. However, with a determined *p*-value of *p* = 0.814 when performing a t-test, no difference could be identified between the age at embolization of later recanalized PAVMs and non-reperfused PAVMs, meaning that the age at embolization does not appear to have any influence on later recanalization. Furthermore, the growth of embolized PAVMs during childhood does not appear to be associated with an increased recanalization rate.

In terms of gender distribution, there is a clear surplus of women in both groups, with a ratio of women to men of 2:1 in patients with recanalization and 2.4:1 in patients without reperfusion. The literature also describes an excess of women with PAVM requiring treatment in adulthood [27]. However, in the literature, it is also described that in newborns and children this ratio is in favor of boys, suggesting that PAVMs requiring treatment in childhood occur more frequently in boys and that this ratio is reversed in adulthood [27]. However, a statistical examination of the gender distribution between the two groups showed with a *p*-value of *p* = 0.678 in the chi-square test that recanalized PAVMs do not occur more frequently in a particular gender.

When analyzing the feeding artery as part of our investigations, a statistically significant difference in the size of the feeding artery between the two groups was demonstrated: at a diameter of 4.4 mm in the recanalized AV shunts and 3.4 mm in the non-reperfused fistulas, a *p*-value of *p* < 0.001 could be determined. Our study, therefore, supports the assumption that an increased diameter of the feeding artery is a risk factor for reperfusion after embolization. In comparison, a study by Shimora et al. found a median of 3.8 mm in patients with reperfusion and a median of 3 mm in non-reperfused patients [21]. Another study from 2005 by Milic et al. showed a diameter of 4.7 mm in reperfused and 3.9 mm in non-reperfused PAVM (*p* < 0.001) [15].

In addition, the proportion of treatment-relevant PAVMs in the various lung lobes was determined. In total, the localization of 263 PAVMs was evaluated, with a proportion of 28.1% in the right lower lobe and 30% in the left lower lobe, showing an emphasis on the lower lobes of the lung. Other studies also describe a proportion of 65% and 69% of therapy-relevant PAVMs in the lower lobes of the lung [12,20], meaning that our investigations also showed an increased occurrence of malformations in the basal parts of the lung.

The following findings were obtained when evaluating the causes of reperfusion: a total of 64 recanalized AV shunts were identified for which angiographic images were available for further assessment. Of these, 52 feeding arteries could be attributed to an inadequate packing density of the implanted coils. In 15 of these 52 AV fistulas, the spiral formation of the implanted coils on the arterial wall was observed, which meant that a high packing density within the vessel could not be achieved and thus no permanent occlusion could be achieved. In comparison, another study by Pollak et al. with a total of 415 examined PAVMs also came to the conclusion that recanalization was most frequently caused by inadequately packed coils [13]. These results show that the packing density of implanted coils is a risk factor for recanalization. On further evaluation of the other fistulas, the dilatation of the feeding artery at the implantation site of the embolization material was observed in an AV shunt, allowing blood to flow past it and the malformation to re-perfuse. There are also isolated reports in the literature of a dilation of the feeding artery at the site of the embolization material, whereby a flow through the reperfused PAVM was also detected [24]. The dilation of the feeding artery with a reperfusion of the PAVM can therefore occasionally occur, advocating for long-term follow-up. Perfusion was detected in two externally embolized PAVMs, which were initially occluded by detachable coils in the aneurysm sac. Subsequently, these were the only two malformations that were embolized by detachable coils. These coils are usually used to treat cerebral aneurysms, although their structure differs from that of pulmonary aneurysms. In addition, some studies have already shown that these coils do not anchor in the wall of the aneurysm and can become denser over time, which can lead to the recanalization of cerebral aneurysms [23,28]. The anchoring of the coils was also not present in the two patients in our examinations and the embolization material was located centrally in the aneurysm sac in both patients so that these PAVMs were found to be reperfused in examinations at our department. In this respect, it is questionable whether detachable coils are suitable for the embolization of PAVMs, as there is always a risk of migration of the embolization material into the systemic circulation due to the lack of sufficient long-term anchoring of the coils. This risk does not exist with the simultaneous extensive embolization of the feeding artery, as in the current study by Mathevosian et al. However, too much healthy lung tissue may also be occluded if the feeding artery is embolized too extensively [29]. Reperfusion could be attributed to the growth in size of the patient’s feeding artery as part of body growth. This has also been reported in individual cases [30], so that this is also a cause for recanalization after therapy. Furthermore, the reperfusion of an AVP 4 was observed after almost six years, although this AV shunt was still occluded at three previous follow-up visits. Individual cases of reperfusion of Amplatzer™ vascular plugs are also known in the literature [24,25], but large-scale studies also show permanent and sufficient occlusion after implantation of Amplatzer™ vascular plugs [31,32,33,34]. In our present study, no reperfusion of an AVP II or of AV shunts embolized from a combination of AVP II or 4 with at least one coil was detected. However, it should be emphasized that Amplatzer™ vascular plugs have not been available for as long as coils since their market launch in 2004 [35]. For this reason, longer follow-up periods should be sought in order to detect any delayed reperfusion of this embolization material. In one case, it can be assumed that reperfusion has occurred due to the corrosion of the implanted tungsten coils. It has already been described several times in the literature that the resorption of the material and even reperfusion can occur over time after the implantation of tungsten objects [36,37,38]. As at least six years elapse between embolization and reperfusion in this case, the corrosion of the material as the cause of reperfusion would be an obvious mechanism. In another patient with three re-embolizations of the same malformation, no obvious cause of reperfusion could be determined either, but it is striking that a re-embolization of the same malformation had to be performed a total of three times. In each case, care was taken to ensure a sufficient number of coils and a high packing density, but recanalization occurred nonetheless over time. In this case, it is worth discussing whether this patient’s multiple recanalization was caused by extraordinarily fibrinolytic activity in the lung.

Upon a closer evaluation of the coils, which had a lower thickness than the Nester^®^ Embolization Coil (Cook Medical) when examining the DSA and corresponded to a total of 45.3% of the total recanalized AV shunts, a correlation between the thickness of the coils and possible recanalization after therapy was discussed. A spiral can be compared to the shape of a cylinder as an example, so that the formula V = ᴫr^2^h applies to the calculation of the volume. The Nester^®^ Embolization Coil from Cook Medical has a thickness of 0.035 inch, resulting in a volume of 86.9 mm^3^ for the regularly used length of 14 cm. There are also microcoils from the same company (Nester^®^ Embolization Microcoil), which are also available in a length of 14 cm and have the same properties as the Nester^®^ Embolization Coil but have a thickness of 0.018 inch. Calculating the volume of these coils results in a value of V = 22.98 mm^3^, which corresponds to approximately a quarter of the volume of the Nester^®^ Embolization Coil. Assuming that the coils are implanted on the same route within the vessel, almost four times the volume can be implanted using double-strength coils, which may be relevant for permanent sufficient occlusion with regard to a high packing density within the feeding artery. Furthermore, the catheters for larger-thickness coils have a higher rigidity than catheters used for implanting microcoils, so that the catheter is only pushed back slightly when large-thickness coils are inserted under pressure, making it easier to achieve a high packing density of the coils. To evaluate if the thickness of coils has an impact on later reperfusion, a two-sided chi-square test as described above has been performed. With a *p*-value of *p* < 0.001, the coils with a lower thickness seem to be associated with a higher recanalization rate after therapy. Nevertheless, this result needs to be viewed critically, since it was not possible to examine the exact embolization material of all patients so we could only estimate the coil thickness of 17 patients visually, as described above.

In the patients with collateral formation after embolization, this could be identified either directly at the first or at the second follow-up appointment after embolization. Thus, reperfusion by collaterals after embolization appears to occur significantly faster than recanalization. In a total of ten of the patients with collateral formation, this was observed between two pulmonary arteries. In one patient, however, the formation of collaterals between the systemic and pulmonary arterial circulation in the sense of aortopulmonary collaterals was detected. These results are consistent with the literature, where collateral formation between two pulmonary arteries is also described much more frequently than reperfusion from the systemic circulation [15,39].

The present study has two limitations. First, due to the retrospective study design, not all parameters could be collected from every patient. For example, the exact reperfusion and follow-up period, the age at embolization and the localization of the treated malformations could not be determined due to a lack of reports for the majority of externally embolized patients. Secondly, not all patients regularly attended the follow-up appointments. In many cases, there was a clear longer period than recommended between the individual follow-up appointments. In this respect, the reperfusion period could not be determined exactly, even in patients with a known embolization date, resulting in a distorted longer reperfusion time. Nevertheless, the values provide information that reperfusion can still occur a long time after embolization and that follow-up checks are essential.

In addition, the results may be influenced by the data of the patients treated externally. First, all of them had at least one reperfused PAVM, meaning that the reperfusion rate of patients treated internally and externally is higher than for the patients only treated internally. Second, coils for embolization at our clinic are predominantly used with a thickness of 0.035 inch in contrast to many patients treated externally who were often embolized by coils with a lower thickness. Due to the fact that these patients had at least one reperfused PAVM, this could lead to a distortion of the results, meaning that the influence of coils with a lower thickness to a later reperfusion is less strong than currently assumed. The follow-up period, the time between embolization and first described reperfusion/re-embolization and the age of the patients could not be determined for all patients treated externally, but there is no distortion through the external patients because these parameters were only analyzed from patients with known data and resulted only in a lower number of total analyzed cases. Beyond that, the gender of patients, the size and number of the feeding arteries and the embolization material of the recanalized arteries could have been determined for all treated patients, so there are not any impacts from the external treatment data on the total analysis.

In summary, life-long, regular follow-up checks should be performed after embolization in order to detect reperfusion at an early stage and avoid further complications. When catheter-based treatment by embolization with coils is performed, a sufficiently high packing density should be ensured, and embolization with Amplatzer™ vascular plugs in combination with coils should be considered as a suitable treatment option for the sufficient closure of PAVMs without a risk of reperfusion. Embolization using detachable coils in the aneurysm sac alone, however, is questionable. Furthermore, this study confirmed that treatment-relevant PAVMs occur more frequently in the basal parts of the lung and that an increased diameter of the feeding artery is a risk factor for subsequent reperfusion.

## Figures and Tables

**Table 1:** MRA sequence parameters for the time-resolved and high-resolution MRA.

MR Angiography Sequence Parameters		
	Time-resolved MRA	High-resolution MRA
Repetition time (TR)	2.7 ms	2.81 ms
Echo time (TE)	1.0 ms	1.07 ms
Flip angle	25	25
Slice thickness	1.5 mm	1.3 mm
Field of view (FOV)	40 × 29 cm	40 × 29 cm
Number of slices	72	160
Number of averages	1	1
Temporal resolution of one data set	3 s	2.2 s
Wrap around body coil (18-channel bodymetric coil)	Yes	Yes
Bandwidth (BW)	±113 kHz	540 kHz

## Data Availability

The data presented in this study are available on request from the corresponding author due to privacy and ethical reasons.
